# Supplementary material for: Virus diversity, wildlife-domestic animal circulation and potential zoonotic viruses of small mammals, pangolins and zoo animals
Source: Nat Commun. 2023 Apr 29;14:2488. doi: 10.1038/s41467-023-38202-4 (PMC10148632; doi:10.1038/s41467-023-38202-4)
Supplement: Supplementary file 1 — Supplementary Information [file 41467_2023_38202_MOESM1_ESM.pdf]

Virus diversity, wildlife-domestic animal circulation and potential zoonotic viruses of small mammals, pangolins and zoo animals

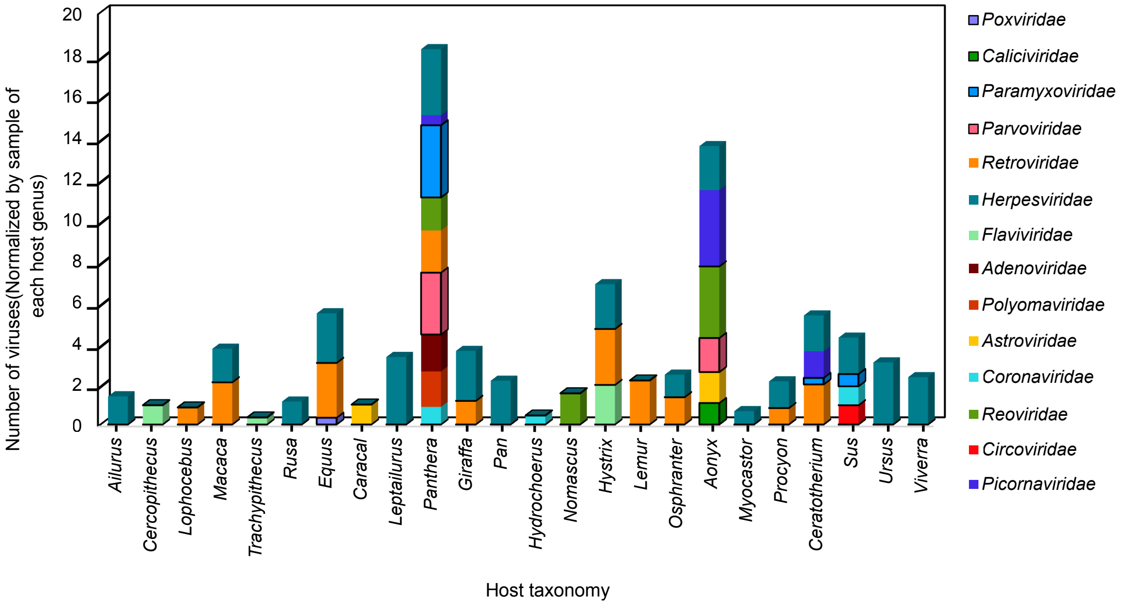

Supplementary Figure 1. **Overview of the diversity and abundance of the viruses identified in zoo animals classified by viral family and host genus.**

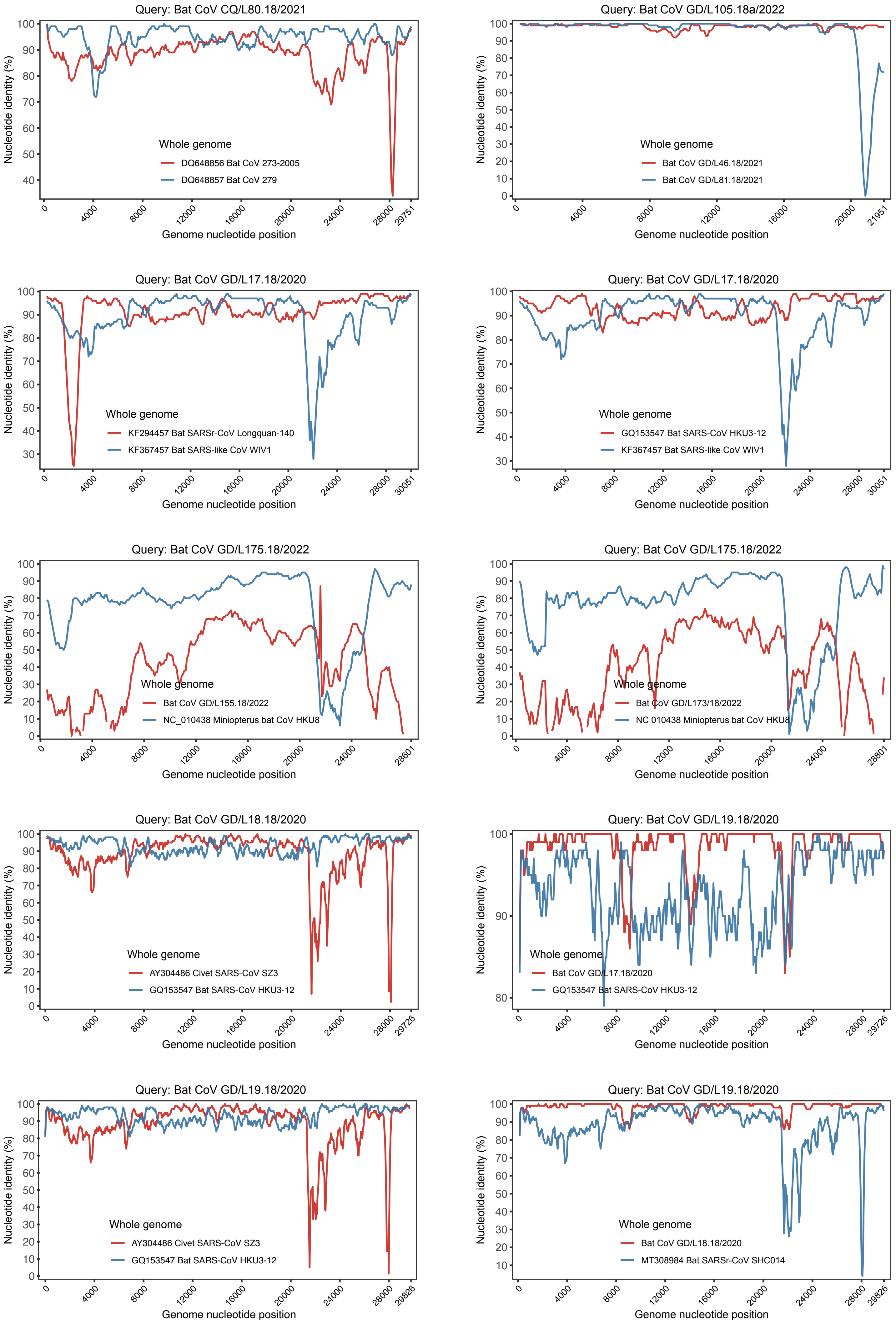
Query: Bat CoV CQ/L80.18/2021
Whole genome
DQ648856 Bat CoV 273-2005
DQ648857 Bat CoV 279
Query: Bat CoV GD/L105.18a/2022
Whole genome
Bat CoV GD/L46.18/2021
Bat CoV GD/L81.18/2021
Query: Bat CoV GD/L17.18/2020
Whole genome
KF294457 Bat SARSr-CoV Longquan-140
KF367457 Bat SARS-like CoV WIV1
Query: Bat CoV GD/L17.18/2020
Whole genome
GQ153547 Bat SARS-CoV HKU3-12
KF367457 Bat SARS-like CoV WIV1
Query: Bat CoV GD/L175.18/2022
Whole genome
Bat CoV GD/L155.18/2022
NC_010438 Miniopterus bat CoV HKU8
Query: Bat CoV GD/L175.18/2022
Whole genome
Bat CoV GD/L173/18/2022
NC 010438 Miniopterus bat CoV HKU8
Query: Bat CoV GD/L18.18/2020
Whole genome
AY304486 Civet SARS-CoV SZ3
GQ153547 Bat SARS-CoV HKU3-12
Query: Bat CoV GD/L19.18/2020
Whole genome
Bat CoV GD/L17.18/2020
GQ153547 Bat SARS-CoV HKU3-12
Query: Bat CoV GD/L19.18/2020
Whole genome
AY304486 Civet SARS-CoV SZ3
GQ153547 Bat SARS-CoV HKU3-12
Query: Bat CoV GD/L19.18/2020
Whole genome
Bat CoV GD/L18.18/2020
MT308984 Bat SARSr-CoV SHC014
Nucleotide identity (%)
Genome nucleotide position

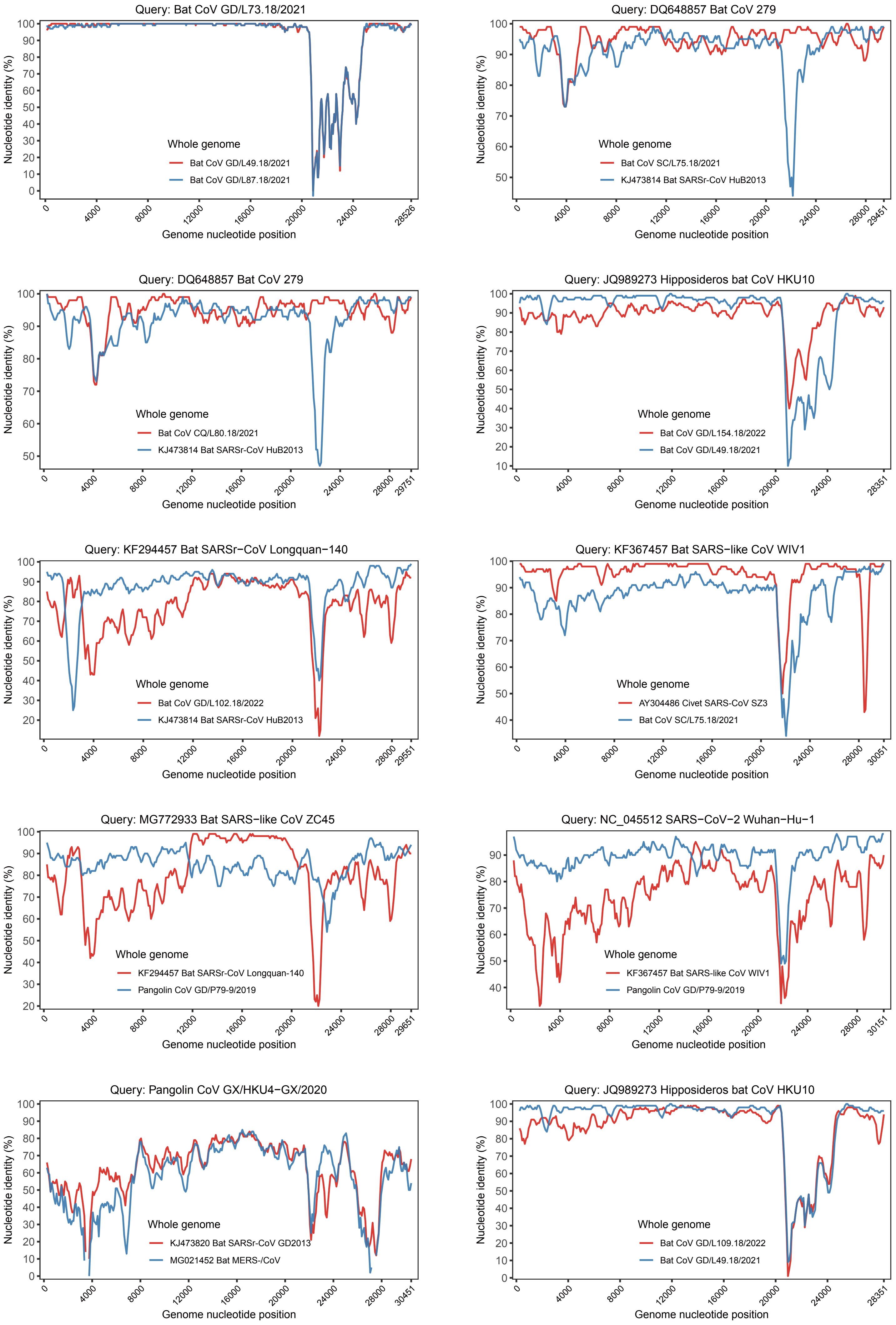

Query: Bat CoV GD/L73.18/2021
Whole genome
Bat CoV GD/L49.18/2021
Bat CoV GD/L87.18/2021
Nucleotide identity (%)
Genome nucleotide position
28526
Query: DQ648857 Bat CoV 279
Whole genome
Bat CoV SC/L75.18/2021
KJ473814 Bat SARSr-CoV HuB2013
Nucleotide identity (%)
Genome nucleotide position
29451
Query: DQ648857 Bat CoV 279
Whole genome
Bat CoV CQ/L80.18/2021
KJ473814 Bat SARSr-CoV HuB2013
Nucleotide identity (%)
Genome nucleotide position
29751
Query: JQ989273 Hipposideros bat CoV HKU10
Whole genome
Bat CoV GD/L154.18/2022
Bat CoV GD/L49.18/2021
Nucleotide identity (%)
Genome nucleotide position
28351
Query: KF294457 Bat SARSr−CoV Longquan−140
Whole genome
Bat CoV GD/L102.18/2022
KJ473814 Bat SARSr-CoV HuB2013
Nucleotide identity (%)
Genome nucleotide position
29551
Query: KF367457 Bat SARS−like CoV WIV1
Whole genome
AY304486 Civet SARS-CoV SZ3
Bat CoV SC/L75.18/2021
Nucleotide identity (%)
Genome nucleotide position
30051
Query: MG772933 Bat SARS−like CoV ZC45
Whole genome
KF294457 Bat SARSr-CoV Longquan-140
Pangolin CoV GD/P79-9/2019
Nucleotide identity (%)
Genome nucleotide position
29651
Query: NC_045512 SARS−CoV−2 Wuhan−Hu−1
Whole genome
KF367457 Bat SARS-like CoV WIV1
Pangolin CoV GD/P79-9/2019
Nucleotide identity (%)
Genome nucleotide position
30151
Query: Pangolin CoV GX/HKU4−GX/2020
Whole genome
KJ473820 Bat SARSr-CoV GD2013
MG021452 Bat MERS-/CoV
Nucleotide identity (%)
Genome nucleotide position
30451
Query: JQ989273 Hipposideros bat CoV HKU10
Whole genome
Bat CoV GD/L109.18/2022
Bat CoV GD/L49.18/2021
Nucleotide identity (%)
Genome nucleotide position
28351

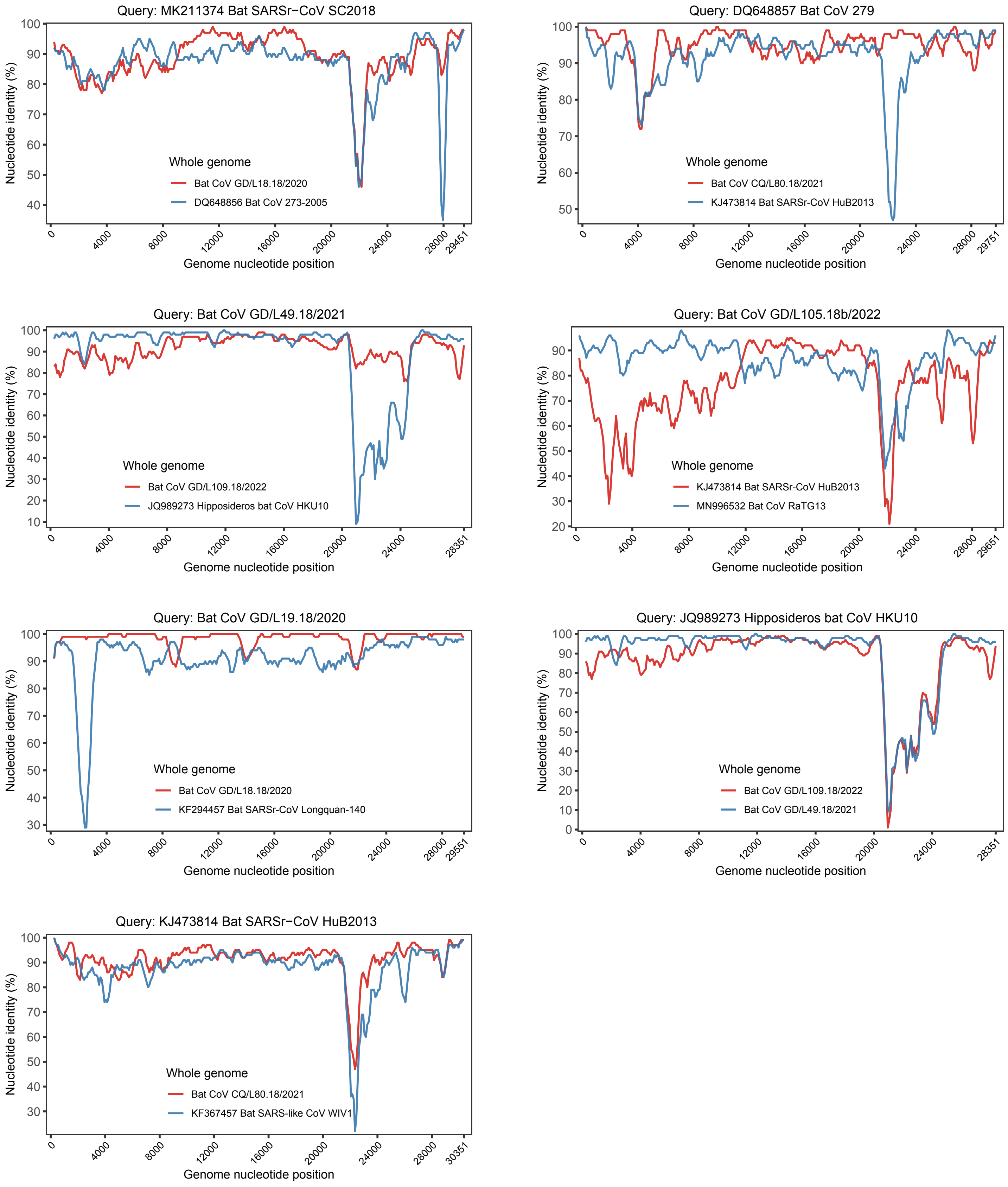

Supplementary Figure 2. **Similarity plot based on the full-length genome sequences of CoVs.** Parameters for the similarity plots are: window, 500 bp; step, 100 bp.

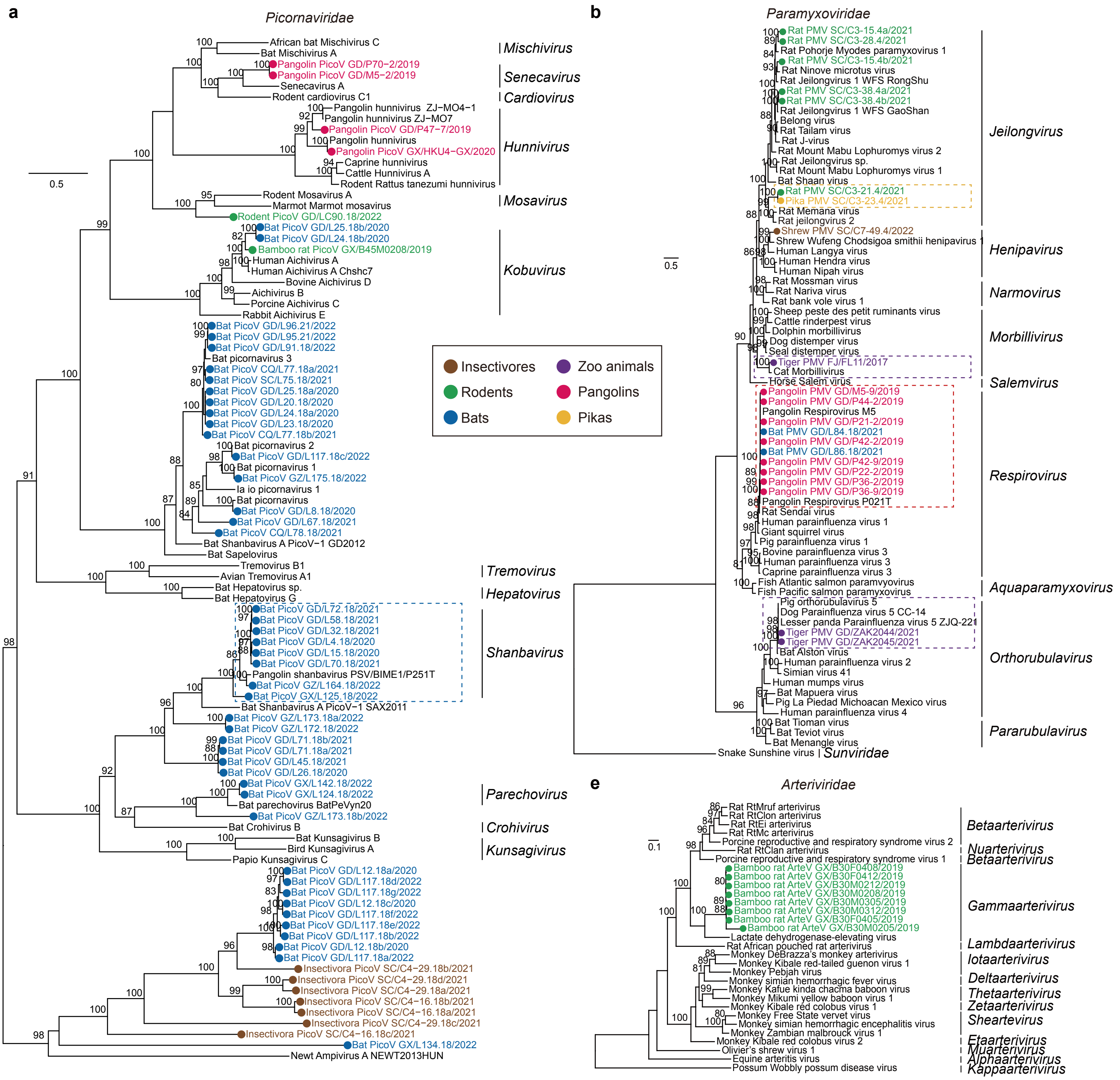

a
Picornaviridae
Mischivirus
African bat Mischivirus C
Bat Mischivirus A
Senecavirus
Pangolin PicoV GD/P70−2/2019
Pangolin PicoV GD/M5−2/2019
Senecavirus A
Cardiovirus
Rodent cardiovirus C1
Hunnivirus
Pangolin hunnivirus ZJ−MO4−1
Pangolin hunnivirus ZJ−MO7
Pangolin PicoV GD/P47−7/2019
Pangolin hunnivirus
Pangolin PicoV GX/HKU4−GX/2020
Caprine hunnivirus
Cattle Hunnivirus A
Rodent Rattus tanezumi hunnivirus
Mosavirus
Rodent Mosavirus A
Marmot Marmot mosavirus
Rodent PicoV GD/LC90.18/2022
Kobuvirus
Bat PicoV GD/L25.18b/2020
Bat PicoV GD/L24.18b/2020
Bamboo rat PicoV GX/B45M0208/2019
Human Aichivirus A
Human Aichivirus A Chshc7
Bovine Aichivirus D
Aichivirus B
Porcine Aichivirus C
Rabbit Aichivirus E
Bat PicoV GD/L96.21/2022
Bat PicoV GD/L95.21/2022
Bat PicoV GD/L91.18/2022
Bat picornavirus 3
Bat PicoV CQ/L77.18a/2021
Bat PicoV SC/L75.18/2021
Bat PicoV GD/L25.18a/2020
Bat PicoV GD/L20.18/2020
Bat PicoV GD/L24.18a/2020
Bat PicoV GD/L23.18/2020
Bat PicoV CQ/L77.18b/2021
Bat picornavirus 2
Bat PicoV GD/L117.18c/2022
Bat picornavirus 1
Bat PicoV GZ/L175.18/2022
Ia io picornavirus 1
Bat picornavirus
Bat PicoV GD/L8.18/2020
Bat PicoV GD/L67.18/2021
Bat PicoV CQ/L78.18/2021
Bat Shanbavirus A PicoV−1 GD2012
Bat Sapelovirus
Tremovirus
Tremovirus B1
Avian Tremovirus A1
Hepatovirus
Bat Hepatovirus sp.
Bat Hepatovirus G
Shanbavirus
Bat PicoV GD/L72.18/2021
Bat PicoV GD/L58.18/2021
Bat PicoV GD/L32.18/2021
Bat PicoV GD/L4.18/2020
Bat PicoV GD/L15.18/2020
Bat PicoV GD/L70.18/2021
Pangolin shanbavirus PSV/BIME1/P251T
Bat PicoV GZ/L164.18/2022
Bat PicoV GX/L125.18/2022
Bat Shanbavirus A PicoV−1 SAX2011
Bat PicoV GZ/L173.18a/2022
Bat PicoV GZ/L172.18/2022
Bat PicoV GD/L71.18b/2021
Bat PicoV GD/L71.18a/2021
Bat PicoV GD/L45.18/2021
Bat PicoV GD/L26.18/2020
Parechovirus
Bat PicoV GX/L142.18/2022
Bat PicoV GX/L124.18/2022
Bat parechovirus BatPeVyn20
Bat PicoV GZ/L173.18b/2022
Crohivirus
Bat Crohivirus B
Kunsagivirus
Bat Kunsagivirus B
Bird Kunsagivirus A
Papio Kunsagivirus C
Bat PicoV GD/L12.18a/2020
Bat PicoV GD/L117.18d/2022
Bat PicoV GD/L117.18g/2022
Bat PicoV GD/L12.18c/2020
Bat PicoV GD/L117.18f/2022
Bat PicoV GD/L117.18e/2022
Bat PicoV GD/L117.18b/2022
Bat PicoV GD/L12.18b/2020
Bat PicoV GD/L117.18a/2022
Insectivora PicoV SC/C4−29.18b/2021
Insectivora PicoV SC/C4−29.18d/2021
Insectivora PicoV SC/C4−29.18a/2021
Insectivora PicoV SC/C4−16.18b/2021
Insectivora PicoV SC/C4−16.18a/2021
Insectivora PicoV SC/C4−29.18c/2021
Insectivora PicoV SC/C4−16.18c/2021
Bat PicoV GX/L134.18/2022
Newt Ampivirus A NEWT2013HUN
0.5
Insectivores
Zoo animals
Rodents
Pangolins
Bats
Pikas
b
Paramyxoviridae
Jeilongvirus
Rat PMV SC/C3-15.4a/2021
Rat PMV SC/C3-28.4/2021
Rat Pohorje Myodes paramyxovirus 1
Rat PMV SC/C3-15.4b/2021
Rat Ninove microtus virus
Rat Jeilongvirus 1 WFS RongShu
Rat PMV SC/C3-38.4a/2021
Rat PMV SC/C3-38.4b/2021
Rat Jeilongvirus 1 WFS GaoShan
Belong virus
Rat Tailam virus
Rat J-virus
Rat Mount Mabu Lophuromys virus 2
Rat Jeilongvirus sp.
Rat Mount Mabu Lophuromys virus 1
Bat Shaan virus
Rat PMV SC/C3-21.4/2021
Pika PMV SC/C3-23.4/2021
Rat Memana virus
Rat jeilongvirus 2
Henipavirus
Shrew PMV SC/C7-49.4/2022
Shrew Wufeng Chodsigoa smithii henipavirus 1
Human Langya virus
Human Hendra virus
Human Nipah virus
Narmovirus
Rat Mossman virus
Rat Nariva virus
Rat bank vole virus 1
Morbillivirus
Sheep peste des petit ruminants virus
Cattle rinderpest virus
Dolphin morbillivirus
Dog distemper virus
Seal distemper virus
Tiger PMV FJ/FL11/2017
Cat Morbillivirus
Salemvirus
Horse Salem virus
Respirovirus
Pangolin PMV GD/M5-9/2019
Pangolin PMV GD/P44-2/2019
Pangolin Respirovirus M5
Pangolin PMV GD/P21-2/2019
Bat PMV GD/L84.18/2021
Pangolin PMV GD/P42-2/2019
Bat PMV GD/L86.18/2021
Pangolin PMV GD/P42-9/2019
Pangolin PMV GD/P22-2/2019
Pangolin PMV GD/P36-2/2019
Pangolin PMV GD/P36-9/2019
Pangolin Respirovirus P021T
Rat Sendai virus
Human parainfluenza virus 1
Giant squirrel virus
Pig parainfluenza virus 1
Bovine parainfluenza virus 3
Human parainfluenza virus 3
Caprine parainfluenza virus 3
Aquaparamyxovirus
Fish Atlantic salmon paramyvovirus
Fish Pacific salmon paramyxovirus
Orthorubulavirus
Pig orthorubulavirus 5
Dog Parainfluenza virus 5 CC-14
Lesser panda Parainfluenza virus 5 ZJQ-221
Tiger PMV GD/ZAK2044/2021
Tiger PMV GD/ZAK2045/2021
Bat Alston virus
Human parainfluenza virus 2
Simian virus 41
Human mumps virus
Bat Mapuera virus
Pig La Piedad Michoacan Mexico virus
Human parainfluenza virus 4
Pararubulavirus
Bat Tioman virus
Bat Teviot virus
Bat Menangle virus
Snake Sunshine virus
Sunviridae
0.5
e
Arteriviridae
Betaarterivirus
Rat RtMruf arterivirus
Rat RtClon arterivirus
Rat RtEi arterivirus
Rat RtMc arterivirus
Porcine reproductive and respiratory syndrome virus 2
Nuarterivirus
Rat RtClan arterivirus
Betaarterivirus
Porcine reproductive and respiratory syndrome virus 1
Gammaarterivirus
Bamboo rat ArteV GX/B30F0408/2019
Bamboo rat ArteV GX/B30F0412/2019
Bamboo rat ArteV GX/B30M0212/2019
Bamboo rat ArteV GX/B30M0208/2019
Bamboo rat ArteV GX/B30M0305/2019
Bamboo rat ArteV GX/B30M0312/2019
Bamboo rat ArteV GX/B30F0405/2019
Bamboo rat ArteV GX/B30M0205/2019
Lactate dehydrogenase-elevating virus
Lambdaarterivirus
Rat African pouched rat arterivirus
Iotaarterivirus
Monkey DeBrazza's monkey arterivirus
Monkey Kibale red-tailed guenon virus 1
Monkey Pebjah virus
Deltaarterivirus
Monkey simian hemorrhagic fever virus
Thetaarterivirus
Monkey Kafue kinda chacma baboon virus
Monkey Mikumi yellow baboon virus 1
Zetaarterivirus
Monkey Kibale red colobus virus 1
Sheartevirus
Monkey Free State vervet virus
Monkey simian hemorrhagic encephalitis virus
Monkey Zambian malbrouck virus 1
Etaarterivirus
Monkey Kibale red colobus virus 2
Muarterivirus
Olivier's shrew virus 1
Alphaarterivirus
Equine arteritis virus
Kappaarterivirus
Possum Wobbly possum disease virus
0.1

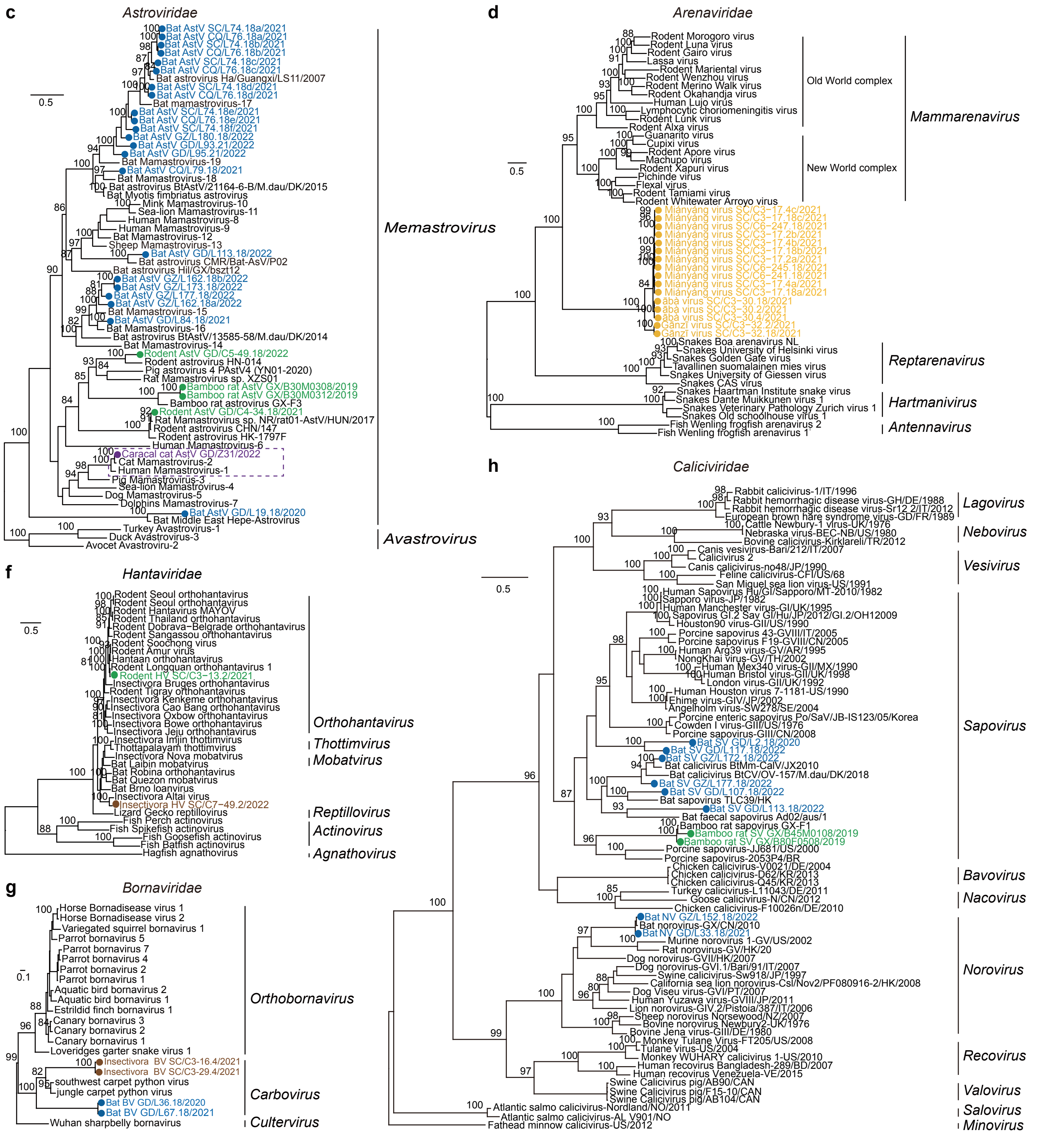

Supplementary Figure 3. **Phylogeny trees of other RNA viral families obtained here, related to Figure 4.** (**a**) *Picornaviridae*, (**b**) *Paramyxoviridae*, (**c**) *Astroviridae,* (**d**) *Arenaviridae*, (**e**) *Arteriviridae*, (**f**) *Hantaviridae*, (**g**) *Bornaviridae* and (**h**) *Caliciviridae*. These trees were constructed using the same methods descried in the figure legend of Figure 4. Virus names are showed in the topologies.

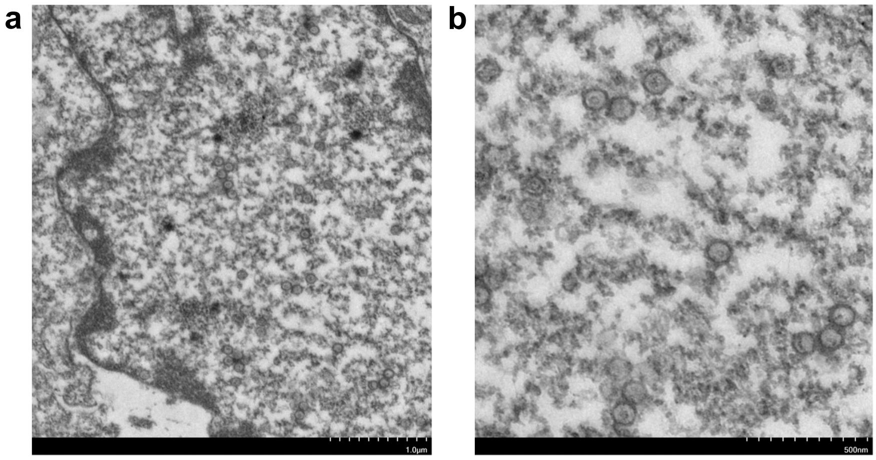

Supplementary Figure 4. **Electron micrograph of PRV particles of PRV FJ/tiger/2015**. (**a**) 1.0 μm. (**b**) 500 nm. These experiments were performed in triplicate independently with similar results.

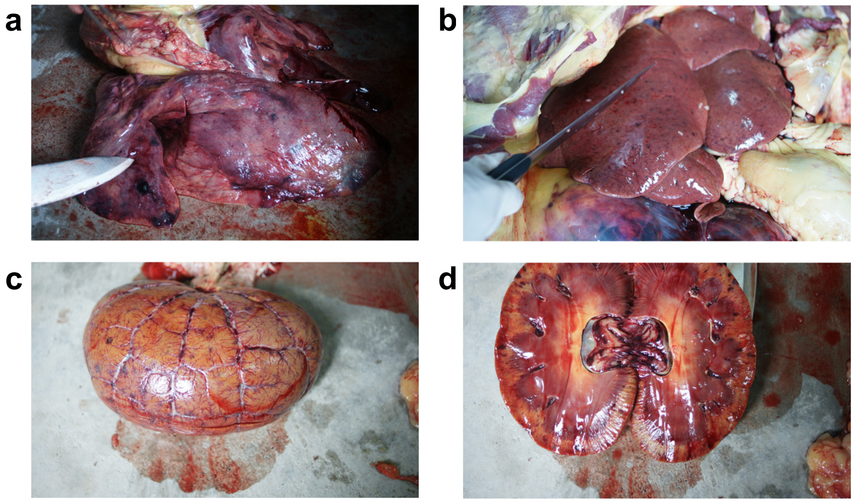

Supplementary Figure 5. **Necropsy, showing pathologic changes in a tiger naturally infected with PRV.** (**a**) Lung presented severe hemorrhages and congestion. (**b**) Liver and kidney (**c** and **d**) show focal hemorrhage.

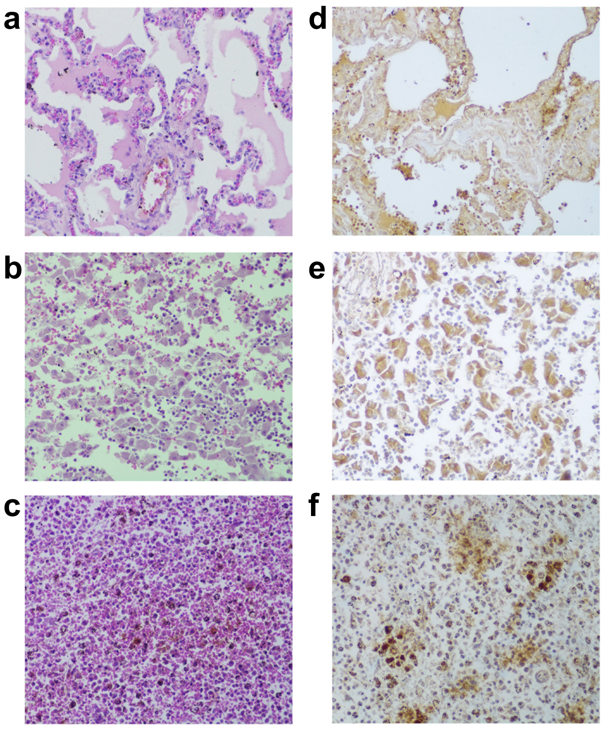

Supplementary Figure 6. **Histopathological changes and immunohistochemical detection of PRV antigen in a PRV infected tiger.** Histopathological changes of the lung (**a**), liver (**b**) and spleen (**c**). Immunohistochemical detection of PRV antigen in the lung (**d**), liver (**e**) and spleen (**f**). Magnification of the images is × 200. These experiments were performed in triplicate independently with similar results.

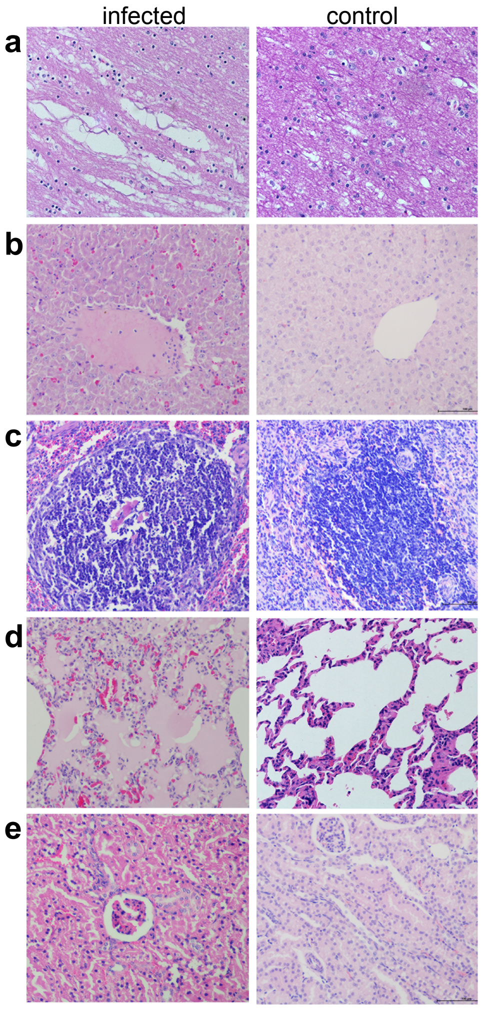

Supplementary Figure 7. **Histopathological changes after PRV challenge in cats.** (**a**) brain; (**b**) liver; (**c**) spleen; (**d**) lung; (**e**) kidney. Magnification of the images is × 200. These experiments were performed in triplicate independently with similar results.

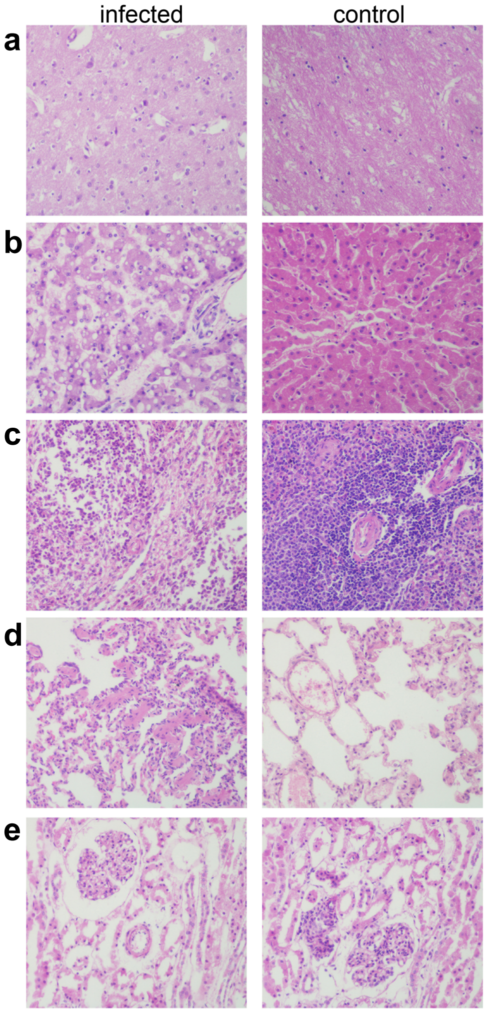

Supplementary Figure 8. **Histopathological changes after PRV challenge in dogs.** (**a**) brain; (**b**) liver; (**c**) spleen; (**d**) lung; (**e**) kidney. Magnification of the images is × 200. These experiments were performed in triplicate independently with similar results.

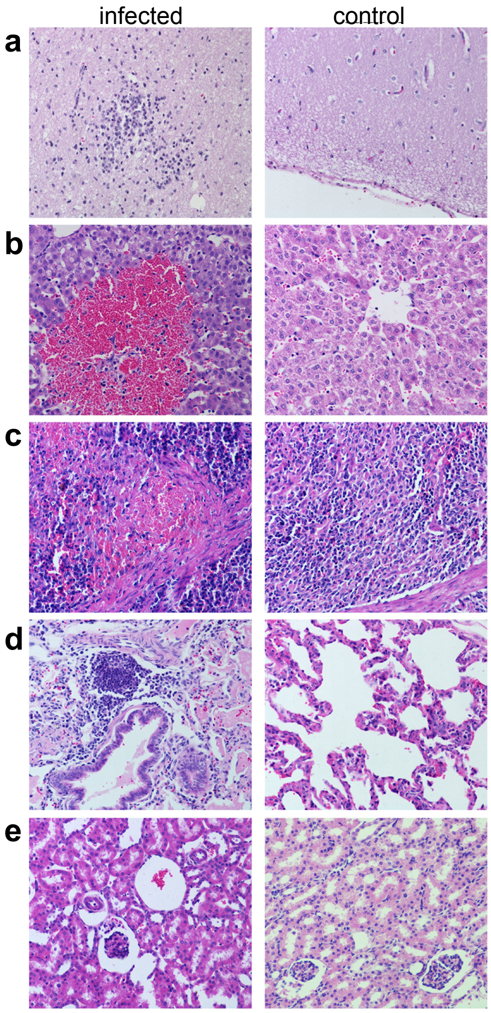

Supplementary Figure 9. **Histopathological changes after PRV challenge in pigs.** (**a**) brain; (**b**) liver; (**c**) spleen; (**d**) lung; (**e**) kidney. Magnification of the images is × 200. These experiments were performed in triplicate independently with similar results.

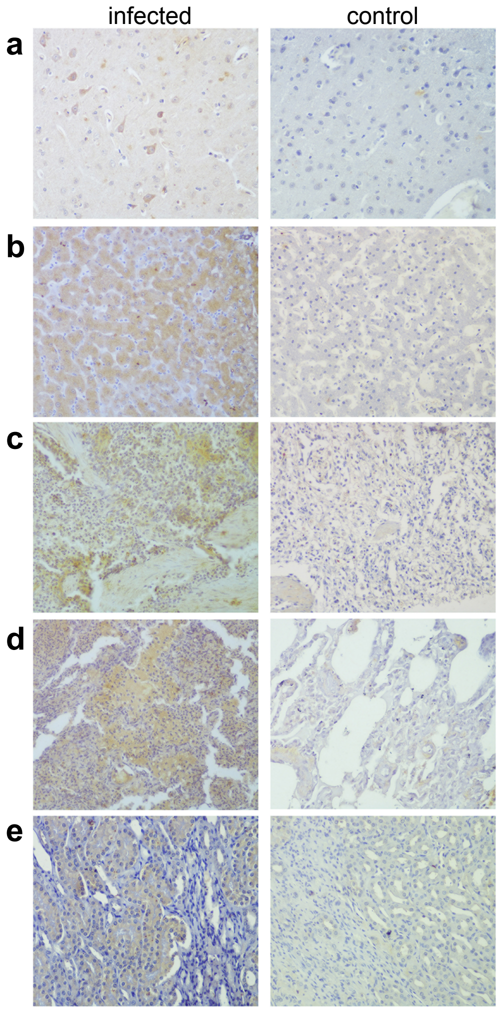

Supplementary Figure 10. **Immunohistochemical detection after PRV challenge in cats.** (**a**) brain; (**b**) liver; (**c**) spleen; (**d**) lung; (**e**) kidney. Magnification of the images is × 200. These experiments were performed in triplicate independently with similar results.

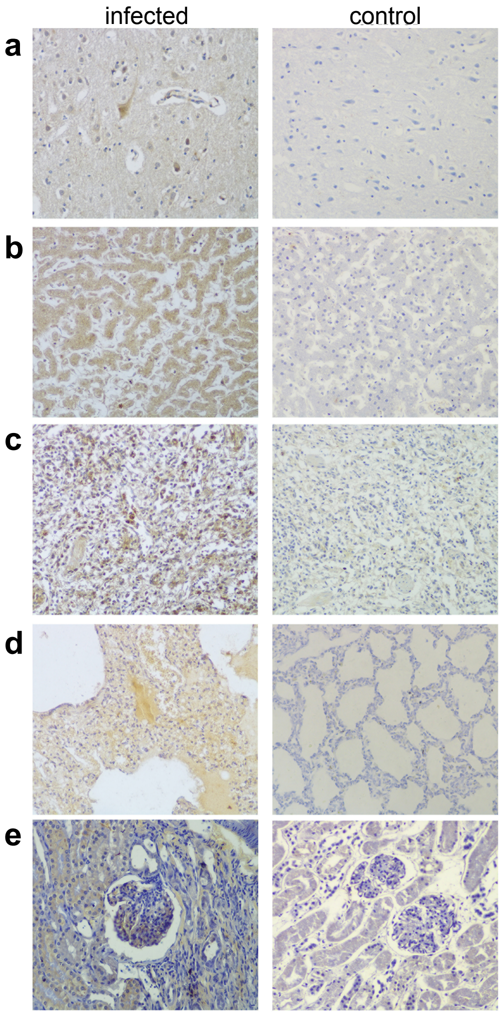

Supplementary Figure 11. **Immunohistochemical detection of PRV antigen after PRV challenge in dogs.** (**a**) brain; (**b**) liver; (**c**) spleen; (**d**) lung; (**e**) kidney. Magnification of the images is × 200. These experiments were performed in triplicate independently with similar results.

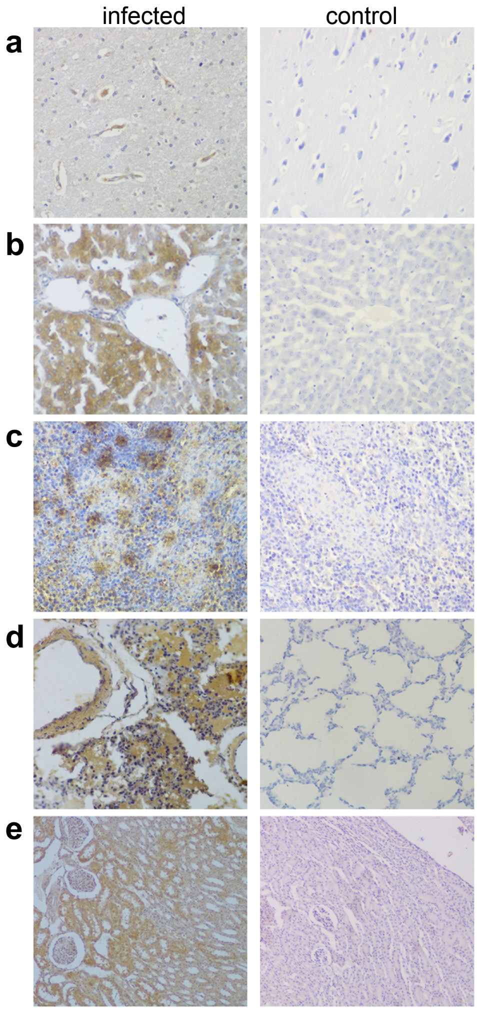

Supplementary Figure 12. **Immunohistochemical detection of PRV antigen after PRV challenge in pigs.** (**a**) brain; (**b**) liver; (**c**) spleen; (**d**) lung; (**e**) kidney. Magnification of the images is × 200. These experiments were performed in triplicate independently with similar results.

SUPPLEMENTARY TABLES

Supplementary Table 1. Summary of viral sequences identified in this study.

	**Near-complete genome**	**Partial genome**	**Total number**
Reported	96	65	161
Unreported	75	92	167
Total number	171	157	328

Supplementary Table 2. Virus distribution in different organs after PRV FJ/tiger/2015 challenge.

**Species** / **Organs**	**Cat**		**Dog**		**Pig**	
	Infected group	Control group	Infected group	Control group	Infected group	Control group
Brain	3/3	0/3	3/3	0/3	3/3	0/3
Heart	2/3	0/3	2/3	0/3	1/3	0/3
Liver	3/3	0/3	3/3	0/3	3/3	0/3
Spleen	1/3	0/3	3/3	0/3	3/3	0/3
Lung	3/3	0/3	3/3	0/3	3/3	0/3
Kidney	1/3	0/3	3/3	0/3	2/3	0/3
lymph gland	2/3	0/3	2/3	0/3	2/3	0/3
