# Supplementary material for: Virus diversity, wildlife-domestic animal circulation and potential zoonotic viruses of small mammals, pangolins and zoo animals
Source: Nat Commun. 2023 Apr 29;14:2488. doi: 10.1038/s41467-023-38202-4 (PMC10148632; doi:10.1038/s41467-023-38202-4)
Supplement: Supplementary file 3 — Description of Additional Supplementary Files [file 41467_2023_38202_MOESM3_ESM.pdf]

Descriptions of Additional Supplementary Files

Supplementary Data 1. Detailed information on the 503 sequencing libraries representing 2175 individual animals.

Supplementary Data 2. The normalized abundance levels (measured by RPM) of each viral species in the different sequencing libraries, related to Figure 1.

Supplementary Data 3. List of viruses identified in this study.

Supplementary Data 4. Confirmation of viruses by RT-PCR and PCR.

Supplementary Data 5. Identification of potential recombination events in CoVs.

Supplementary Data 6. Representative virus strains of each viral family used for phylogenetic analyses.
