# Supplementary material for: Virus diversity, wildlife-domestic animal circulation and potential zoonotic viruses of small mammals, pangolins and zoo animals
Source: Nat Commun. 2023 Apr 29;14:2488. doi: 10.1038/s41467-023-38202-4 (PMC10148632; doi:10.1038/s41467-023-38202-4)
Supplement: Supplementary file 10 — Reporting Summary [file 41467_2023_38202_MOESM10_ESM.pdf]

nature portfolio

Corresponding author(s): Chan Ding, Yi Guan, Changchun Tu, Yongyi Shen

Last updated by author(s): Apr 3, 2023

Reporting Summary

Nature Portfolio wishes to improve the reproducibility of the work that we publish. This form provides structure for consistency and transparency in reporting. For further information on Nature Portfolio policies, see our Editorial Policies and the Editorial Policy Checklist.

Statistics

For all statistical analyses, confirm that the following items are present in the figure legend, table legend, main text, or Methods section.

n/a	Confirmed	
☐	☒	The exact sample size (*n*) for each experimental group/condition, given as a discrete number and unit of measurement
☒	☐	A statement on whether measurements were taken from distinct samples or whether the same sample was measured repeatedly
☒	☐	The statistical test(s) used AND whether they are one- or two-sided<br>*Only common tests should be described solely by name; describe more complex techniques in the Methods section.*
☒	☐	A description of all covariates tested
☒	☐	A description of any assumptions or corrections, such as tests of normality and adjustment for multiple comparisons
☒	☐	A full description of the statistical parameters including central tendency (e.g. means) or other basic estimates (e.g. regression coefficient) AND variation (e.g. standard deviation) or associated estimates of uncertainty (e.g. confidence intervals)
☒	☐	For null hypothesis testing, the test statistic (e.g. *F*, *t*, *r*) with confidence intervals, effect sizes, degrees of freedom and *P* value noted<br>*Give P values as exact values whenever suitable.*
☒	☐	For Bayesian analysis, information on the choice of priors and Markov chain Monte Carlo settings
☒	☐	For hierarchical and complex designs, identification of the appropriate level for tests and full reporting of outcomes
☒	☐	Estimates of effect sizes (e.g. Cohen's *d*, Pearson's *r*), indicating how they were calculated

Our web collection on statistics for biologists contains articles on many of the points above.

Software and code

Policy information about availability of computer code

Data collection	no software was used
Data analysis	Fastp v0.23.1; BWA-MEM v0.7.17; Megahit v1.2.9; BLASTn v2.7.1; DIAMOND v0.8.28.90; Bowtie 2 v2.4.4; TaxonKit v0.2.4; coronaSPAdes v3.15.0; Geneious v2021.2.2; Samtools v1.9; MAFFT v7.407; MEGA v7.0.26; trimAl v1.4.rev15; IQ-Tree v2.0.3; Simplot v3.5.1; RDP v4.97.

For manuscripts utilizing custom algorithms or software that are central to the research but not yet described in published literature, software must be made available to editors and reviewers. We strongly encourage code deposition in a community repository (e.g. GitHub). See the Nature Portfolio guidelines for submitting code & software for further information.

Data

Policy information about availability of data

All manuscripts must include a data availability statement. This statement should provide the following information, where applicable:

- Accession codes, unique identifiers, or web links for publicly available datasets
- A description of any restrictions on data availability
- For clinical datasets or third party data, please ensure that the statement adheres to our policy

Sequence reads generated in this study are available from the NCBI Sequence Read Archive (SRA) database under BioProject accession number PRJNA901878. The complete genome sequences of five tiger PRVs, one porcupine PRV, one lion parvovirus, one pangolin pestivirus have been deposited into GenBank under accession numbers OP727800-OP727805, OP745049 and OP868576 . Assembled sequences were deposited into GenBank under accession numbers OP094593-OP094596, OP860308-OP860416, OP930871-OP930878, OP950229-OP950235, OQ236110-OQ236157, OQ297692-OQ297732, OQ348136-OQ348167, OQ363494-OQ363515,

OQ363750-OQ363806, OQ451885-OQ451890, OP094598, OP094601, OP785141, OP946514, OP971514, OQ316388 and OQ476762. Alignment files and tree files have been deposited in Zenodo (https://zenodo.org/record/7668714#.ZCmdJnZBzIU). The website of the SILVA database is http://www.arb-silva.de.

Human research participants

Policy information about studies involving human research participants and Sex and Gender in Research.

Reporting on sex and gender	NA
Population characteristics	NA
Recruitment	NA
Ethics oversight	NA

Note that full information on the approval of the study protocol must also be provided in the manuscript.

Field-specific reporting

Please select the one below that is the best fit for your research. If you are not sure, read the appropriate sections before making your selection.

[x] Life sciences [ ] Behavioural & social sciences [ ] Ecological, evolutionary & environmental sciences

For a reference copy of the document with all sections, see nature.com/documents/nr-reporting-summary-flat.pdf

Life sciences study design

All studies must disclose on these points even when the disclosure is negative.

Sample size

For metatranscriptome sequencing, because different individuals may carry different viruses, all available samples were used in this study to obtain as much virus data as possible. Tissues, blood, feces or pharyngeal/anal swabs of 1497 bats, 363 rodents, 58 pikas, 18 pangolins, 45 insectivorous animals and 194 zoo animals were used.
To determine the pathogenicity and the potential zoonotic potential of the tiger-isolated PRV (FJ/tiger/2015), cats, dogs and pigs were used. For each animal species, six animals that were negative to PRV were divided into the infected group (three animals) and the control group (three animals). To determine the pathogenicity of the pangolin pestivirus (strain P42), mice, rats, guinea pigs, rabbits and pigs were chosen. Six animals for each species were divided into the infected group (three animals) and the control group (three animals). For these experiments, both the infected group and the control group had three animal individuals for each experiment. Therefore, each experiment had three repetitions to reduce experimental errors.
To determine the pathogenicity of the FPV isolated from a lion, four cats that were negative to FPV were divided into the infected group (three animals) and the control group (one animal). In this experiment, the infected group had three animal individuals, and thus had three repetitions to reduce experimental errors. Considering that the PRV infection experiment showed that the three cats in the control group did not show any pathological changes, in order to reduce the number of sacrificed animals, a cat was used as the control group in this experiment.

Data exclusions

No data were excluded

Replication

For each experimental infections, three animals were used and had similar results. qPCR was performed to detect their infection of viruses. Known positive and negative controls were used throughout, and all assays were performed in triplicate. Transmission electron microscopy and histopathological examination were also performed in triplicate to verify the reproducibility of the experimental findings.

Randomization

For experimental infections, animals were divided into the infected and the control groups randomly.

Blinding

Animals in the infected group and the control group were in different cages. One group of investigators inoculated the animals of the infected group with viruses, therefore, they were not blinded to group allocation. The tissues or swabs of these animals were collected and numbered by this group of investigators. Viral DNA/RNA was detected from these numbered samples by another groups of the investigators who were blind to group allocation. Therefore, the first group of investigators were not blind to group allocation did not influence our results.

Reporting for specific materials, systems and methods

We require information from authors about some types of materials, experimental systems and methods used in many studies. Here, indicate whether each material, system or method listed is relevant to your study. If you are not sure if a list item applies to your research, read the appropriate section before selecting a response.

Materials & experimental systems

n/a	Involved in the study
☒	☐ Antibodies
☐	☒ Eukaryotic cell lines
☒	☐ Palaeontology and archaeology
☐	☒ Animals and other organisms
☒	☐ Clinical data
☒	☐ Dual use research of concern

Methods

n/a	Involved in the study
☒	☐ ChIP-seq
☒	☐ Flow cytometry
☒	☐ MRI-based neuroimaging

Eukaryotic cell lines

Policy information about cell lines and Sex and Gender in Research

Cell line source(s)	MDBK, ATCC, VA, United States;<br>VERO-E6, ATCC, VA, United States;<br>PK-15, ATCC, VA, United States;<br>A549, ATCC, VA, United States;<br>F81, KCB, Kunming, China.
Authentication	STR DNA Profiling
Mycoplasma contamination	All cell lines tested negative for mycoplasma contamination.
Commonly misidentified lines (See ICLAC register)	NA

Animals and other research organisms

Policy information about studies involving animals; ARRIVE guidelines recommended for reporting animal research, and Sex and Gender in Research

Laboratory animals	Mouse C57BL/6, 6 weeks, female;<br>Rat, 6-8 weeks, female;<br>Guinea pig, 6-8 weeks, female;<br>Rabbit, 3 months, female;<br>pig, 2-3 months, female;<br>cat, 6 months for PRV infections, 4-6 weeks for FPV infections, female;<br>dog, 6 months, female.<br>The housing conditions for mice include a temperature range of 20-24°C with 40%-60% humidity, and a 12-hour light/12-hour dark cycle.
Wild animals	For metatranscriptome sequencing, rodents, pikas and insectivorous were collected by traps. They were killed by traps. And their tissues were collected and kept in RNAlater solution (Thermo Fisher Scientific Co.). Then, these samples were placed in a biosafety transport box with dry ice, transported to a P2 laboratory, and used for metatranscriptome sequencing.<br>Bats were collected by mist nets. Then, anal swabs and faeces were collected. After that, they were released in the same region. This work is part of the "fauna of Guangdong province". So a few of them were euthanized using isoflurane and used for making specimens. For these individuals, their tissues were collected. These samples were placed in a biosafety transport box with dry ice, transported to a P2 laboratory, and used for metatranscriptome sequencing.<br>Pangolins were confiscated by Customs and the Department of Forestry of Guangdong Province. These animals were initially sent to Guangzhou wildlife rescue center. Despite exhaustive rescue efforts, all of the pangolins eventually died. Tissues were collected after they died.<br>For zoo animals, the feces were collected by the keepers. Blood and swabs were collected during physical examination of animals. While tissues were collected by veterinarians when dissecting dead animals to find the cause.<br>Scientific name of all of the animals were listed in Supplementary Data 1.xlsx. Their ages were unknown.
Reporting on sex	For metatranscriptome sequencing, both male and female were collected. Samples were pooled according to species, location, and tissue for subsequent RNA extraction and library construction. The purpose of this study is to reveal the viruses carried by animals. There is no evidence that the viruses carried by females and males are different, so the gender factor is not considered.<br>For experimental infections, only females were used. There is no evidence that PRV and FPV have different pathogenicity to female and male animals. In order to reduce the number of animals used, only female animals were selected.
Field-collected samples	Tissues, blood, feces or pharyngeal/anal swabs were stored at -80 °C until metatranscriptome sequencing.
Ethics oversight	All experimental procedures were performed in accordance with animal ethics guidelines and approved by the Animal Care and Use Committee of Longyan University (LY2022003L)

Note that full information on the approval of the study protocol must also be provided in the manuscript.
